# Women and Children Workers

**Published:** 1920-01-31

**Authors:** 


### WOMEN AND CHILDREN WORKERS.

IT is always good to fortify ourselves by comparison with other nations, because the English are probably the keenest of national self-critics, one of the consequences being that those who do not understand our ways are too ready to take us at our own valuation. Last week we were able to show that we can still give America points on the health of schoolchildren. This week we have an interesting parallel between British and Japanese labour conditions, drawn up by the British Vice-Consul at Osaka, who declares that Japan is a century behind us.

A feature of Japanese industry is the quantity of female and child labour. The spinning and weaving companies employ 100,000 girls, of whom a considerable number are children, as against a quarter that number of men. Similarly in weaving of all kinds, the number of women working in 1912 was 133,000 in factories and 205,000 in cottages, while the number of men was only 15,000 and 11,000 respectively. It would be interesting to have a statement on the effect of these conditions on maternity and infant welfare. The Japanese are popularly regarded as a virile race, and in fact have given proof of it. The average Jap is strong and well developed, and the workmen works long hours with little leisure. Labour is cheap, plentiful, and inefficient, largely due to the competition of women and child labour, and it is not well organised. It is to be hoped that Japan's adhesion to some of the important principles laid down at the International Labour Conference in Washington last autumn may bring her better economic conditions and lives of greater leisure.

